# Win/win partnerships between Geneva health-related institutions and caregivers of people with dementia: a descriptive cross-sectional study

**DOI:** 10.1186/s12889-019-7014-8

**Published:** 2019-06-07

**Authors:** Marie-Conception Leocadie, Hélène Lefebvre, Monique Rothan-Tondeur

**Affiliations:** 1 School of Health Sciences, HES-SO University of Applied Sciences and Arts Western Switzerland, Avenue de Champel 47, 1206 Geneva, CH Switzerland; 2 Emérites professor for nursing, Montréal University, Québec, Canada; 3 University Paris 13, Sorbonne Paris Cite, Nursing Sciences Research chair, Laboratory Educations and Health Practices (LEPS), (EA 3412), UFR SMBH, F-93017 Bobigny, France; 4 APHP, Nursing Sciences Research chair Paris, Paris, France

**Keywords:** Caregivers, Partnership, Collaboration, Public heath

## Abstract

**Background:**

In the context of an ageing population and an increase in the appearance of chronic diseases, the commitment of caregivers makes it possible for people confronted with disease to remain at home. Over time, they need support to overcome their difficulties. They also show a need for recognition for their participation in the economic maintenance of the health system. To promote this support, so-called “win/win” partnerships are envisaged. Research is needed to identify the building blocks of an innovative intervention.

**Methods:**

A cross-sectional descriptive study was carried out with health institutions in the canton of Geneva to identify the proportion of institutions with a positive opinion on partnership with caregivers. It has also identified potential partnerships with caregivers of people facing dementia and possible compensation in exchange for the provision of their skills. Descriptive statistics are presented according to their frequencies and relative percentages (categorical variables), as well as by their mean, standard deviation and median (continuous variables). Logistic regression models were used to assess the factors associated with a favorable opinion towards win/win partnerships.

**Results:**

The proportion of executives of health-related institutions with a positive opinion of partnership with caregivers is high: 74.7% (95% CI: 64.8–83.1%). Several types of potential partnerships have been identified between health institutions and caregivers. Areas in which certain activities have been identified as being able to be carried out by caregivers include governance, care, provision of services, accompaniment and support, training and research. Types of compensation for caregivers have also been highlighted.

**Conclusion:**

This study shows that some areas activities of health facilities in the canton of Geneva could be the subject of win-win partnerships with caregivers of people with dementia. Positive view of health executives on partnership with caregivers is encouraging. In the future, innovative projects can emerge to meet the needs of each party.

**Electronic supplementary material:**

The online version of this article (10.1186/s12889-019-7014-8) contains supplementary material, which is available to authorized users.

## Background

In the context of a shortage of health personnel announced by the World Health Organization (WHO) (12.9 million by 2035) [1] and demographic changes that favour an increase in the incidence of chronic diseases, such as Alzheimer’s disease and other types of dementia, contribution of caregivers is essential.

Nevertheless, the overburdening of day-to-day activity causes caregivers to become exhausted, socially isolated and financially challenged. They also develop physical and psychological health problems related to “feeling of burden” [2–6]. The feeling of burden is defined as “all the physical, psychological, emotional, social and financial consequences borne by carers” [7]. Faced with this situation, caregivers’ needs are based on information, training, recognition, coordination of care and mainly, respite [8, 9].

Respite interventions have shown to provide support which is valued by caregivers. They are prevalent and varied. Nevertheless, caregivers do not systematically access respite devices because the services provided do not always answer to their needs. The dissatisfaction of caregivers is associated with many factors such as personal conflicts, organization, finances and their interaction with the health staff [10]. On the other hand, health institutions are commissioned by the political authorities to set up relevant and effective support mechanisms to help caregivers.

In order to provide different solutions adapted to caregivers’ needs, it is necessary to collect data. A new paradigm of partnership is to be explored, so that a win/win partnership between caregivers and health institutions can answer both the caregivers needs as well as the needs of the health institutions. This could be a potential key to meeting their needs, including the need for respite.

This measure may also allow them to overcome difficulties related to lack of activity outside their caregiving. It could reduce the risk of isolation and feeling of burden, as well as increase their sense of competence, recognition and well-being.

Several researchers have already initiated research projects to promote partnerships between patients and health-related institutions. For example, the research team of the University of Montreal designed the “Montreal Model” [11] which integrates the individual skills of patients and/or their families within the different strata and domains of the health system (governance, care, health policy development, teaching, research). French research has put into practice the contribution of “teaching patients” to residents in general medicine who have recognized that this method has allowed for the acquisition of skills [12].

Moreover, the participation of actors in the health system will make it possible to improve the safety and quality of care as a whole. These multiple initiatives are supported by the fact that research has shown that scientific expertise must be nurtured through evidence-based medicine and knowledge-based medicine, among others, by patients and their families [13]. According to Yves Charpak in 2017, citizens who get involved with public health care issues as well as their own health care issues is based around how they position themselves. Citizens have duties but also rights that they want to assert when making decisions not only about public health issues but mostly their own health issues [14]. Other initiatives have been implemented in the United States [15], Great Britain [16] and France [17]. However, patient/family involvement initiatives based on the decisions and functioning of health-related institutions, remain anecdotal, according to Charpak [14]. Unlike other studies, the Hestia program conducted in Geneva, considers the need for caregivers to be acknowledged. In exchange for their competences, caregivers receive some respite free of charge which literature has shown to be their greatest need [18]. Indeed, according to Honneth in 2013, the recognition of society for services rendered is paramount for the psychological stability of citizens [19]. This is the case for family caregivers who, in 2014, thanks to their activities, saved Switzerland’s healthcare system 3.4 billion CHF in costs. To enable the implementation of such a project, it is important to be able to answer several questions. The objectives of this study were to find out what percentages of executives in health-related institutions are favourable to establishing partnership projects with caregivers, as well as which activities related to the functioning of health-related institutions could be entrusted to caregivers in exchange for a free respite device.

## Methods

### Study design

This is a descriptive cross-sectional study of health-related institutions in the canton of Geneva. This study identifies activities that could be undertaken not only by staff within various health institutions but also with the caregivers themselves.

### Settings

This study was conducted among various health institutions in the canton of Geneva: home care settings, nursing homes, hospitals, training centres and associations. Of the 71 health-related institutions that were invited to participate in our survey through postmail, a convenience sample of 21 centres replied positively. They are linked to the support of caregivers caring for patients confronted with Alzheimer’s disease or other forms of dementia. Services not related to the population concerned were not included in the study.

### Study population

In this study, the framework of health-related institutions linked with caregiving to patients confronted with Alzheimer’s disease, or other forms of dementia, were included. Institutional staff who had no connection with the population concerned were not included in the study.

In addition to maximize the responses to our survey, a reminder was scheduled 15 days after the first mailing.

### Sample size

In an arbitrary way and based on feasibility, the minimum percentage of participation desired was 50% of the 188 executives identified in the services participating in the study. Due to having no baseline study by which to calculate a standard deviation sample and appropriate confidence level, the Slovin formula was used for this study. After applying a margin of error of 5% and a correction factor, the minimum number of participants was determined to be 74.

### Data sources/measurement

This study was conducted with a survey which was inspired by the Montreal Model. We found areas of partnership within specific services of health-related institutions: activities related to organization, governance, care, service, accompaniment, support, training and research. The questionnaire consists of four (Additional file 1): the design of an anonymized identification number, sociodemographic data of the institution where the participants practice, description of the activities of the institution where the participants practice and perceptions of a possible partnership with caregivers in exchange for a respite device.

After the questionnaire was designed, three levels of testing were performed:

- First level: evaluation by experts. The survey was submitted for critical review with a pilot committee made up of experts in public health, medicine, education and support of caregivers. They assessed the relevance of the questions and their understanding.
- Second level: evaluation by 10 naive evaluators to evaluate the comprehension of the questionnaire.
- Third level: testing of the online procedure with 10 team leaders then utilising the statistical analysis tool.

After the survey was tested and the agreement of participating institutions was obtained, a link providing access to the survey was sent to the respondents. To ensure the anonymity of participants, a code was assigned. A response time of 15 days was granted. This time was needed for participants to discuss with their care teams and gather the opinions of each member. In addition, to maximize the return of the questionnaires, a reminder was scheduled 15 days after the first mailing.

### Variables

The main outcome was to assess the percentage of cadres of health-related institutions who are in favour of a partnership project. The secondary outcome was to count in percentage the activities related to the functioning of health-related institutions that could be entrusted to caregivers in exchange for a respite device.

### Statistical methods

First, categorical data was described in relation to frequencies and relative proportions overall as well as medical versus non-medical institutions. Continuous variables were be described by their mean, standard deviation (SD) and median overall and by medical versus non-medical institutions. Then, we compared specific answers between medical and non-medical institutions by applying a chi-squared test or Fischer’s exact test according to the conditions of application. We presented associations by odds ratios and their 95% confidence intervals (95% CI). All analyses were performed using STATA IC 15.0 software.

### Ethical consideration

The research protocol was approved by the cantonal research ethics commission; the following number was assigned to the study: Req-2017-00211. A letter of information and consent form for participation in the study were sent by e-mail to the participants. The study modalities, possibilities of withdrawal and the guarantee of anonymity were stipulated. The return of the completed questionnaire constituted proof of the consent granted by the participant.

## Results

### Description of institutions

A total of 95 executives out of 188 from 21 health-related institutions in the canton of Geneva participated in this study: 2 initial training institutions, 1 cantonal hospital, 1 clinic, 12 social medical institutions, 3 home care institutions and 2 associations. Descriptive data identified the type, status, mission and activities (see Table 1).

**Table 1:** Description of institutions and their activities

Variables	*N* (%)
Type of institution	95
Medical and social establishment	12 (12.6)
Home care facility	42 (44.2)
Training institute	16 (16.8)
Patient and family association	1 (1.1)
Hospital/clinic	24 (25.3)
Status of the establishment	
Public	85 (89.5)
Private	10 (10.5)
Main mission of the establishment	
Education/training	16 (16.8)
Care and services	78 (82.1)
Support, advice and guidance	1 (1.1)
Existing activities in the institutions	*N* = 94
Activities related to organizational matters	
Ethics committee	57 (60.6)
Management fee	53 (56.4)
Commission to support a specific population	20 (21.3)
Welcoming newcomers/patients	32 (34.0)
None	2 (2.1)
Don’t know	8 (8.5)
Not applicable to my institution	9 (9.6)
Activities related to the provision of care	
Respite care	50 (52.6)
Nursing care	82 (86.3)
Care seminars	72 (75.8)
Patient/family needs assessment	76 (80.0)
Implementation of patient/family care plans	75 (79.0)
Care coordination	73 (76.8)
Analysis of complex care practice	69 (72.6)
Announcement of a diagnosis	23 (24.2)
Health education/therapeutic education	69 (72.6)
None	1 (1.1)
Don’t know	1 (1.1)
Not applicable to my institution	8 (8.4)
Activities related to the service offer	
Animations	43 (45.3)
Socio-cultural activities	29 (30.5)
Practical help	58 (61.1)
Accompaniment (race …)	61 (64.2)
One-time call for help	27 (28.4)
Meal provision	54 (56.8)
None	5 (5.3)
Don’t know	3 (3.2)
Not applicable to my institution	12 (12.6)
Activities related to training	
Learning through theoretical education	88 (92.6)
Learning through practical teaching	79 (83.2)
Learning through teaching using simulation	29 (30.5)
Learning through practical internships	65 (68.4)
Learning through patients/expert families	20 (21.1)
Initial training of carers	61 (64.2)
Postgraduate training for carers	63 (66.3)
None	0 (0)
Don’t know	1 (1.1)
Not applicable to my institution	2 (2.1)
Research activities	
Design of research protocols	39 (41.5)
Fundraising for the project	33 (35.1)
Request to the ethics committee	40 (42.6)
Research coordination sessions	25 (26.6)
Participation of scientific days	26 (27.7)
None	8 (8.5)
Don’t know	22 (23.4)
Not applicable to my institution	7 (7.5)
Accompaniment and support activities	
Support sessions	41 (43.2)
Advice to patients/families	61 (64.2)
Coordination/orientation of care pathways	54 (56.8)
Help with administrative procedures	55 (57.9)
Family relief/respite	54 (56.8)
None	5 (5.3)
Don’t know	4 (4.2)
Not applicable to my institution	9 (9.5)
Specific services for family caregivers	
Currently, your institution offers services for caregivers	31 (32.6)
Among institutions providing services for helping pockets, average number (± standard deviation, median)	2.6 (±2.8, 1)

Participants in home care facilities were the most likely to participate in the study (44.2%). The institutions that participated in the study were mostly public (89,5%). Their missions were mainly focused on care and services to the population (82.1%). The majority of activities identified in the field of organization and governance were linked to the ethical commissions (60,6%) and to the various management fee (56,4%). Regarding the supply of care, apart from activities related to the announcement of a diagnosis and respite/respite care, the other types of care are represented between 69 and 82%. The most prevalent service offer in health-related institutions was patient support (64.2%), followed by practical help (61.1%) and meal delivery (56.8%). Activities related to training were widely present in the various health-related institutions. Learning via theoretical courses was primarily present (92.6%), followed by learning via practical lessons (83.2%), then learning via practical training (68.4%) followed by postgraduate and initial health training (66.3, 64.2%). Activities related to research were generally the least referenced. Accompaniment and support activities were also represented, with counselling of patients/families (64.2%), coordination/orientation of care pathways (56.8%), family respite (56.8%), help with administrative procedures (57.9%) and the organization of support sessions (43.2%).

In addition, the participants listed secondary activities at their institution (see Table 1).

### Partnership agreement with caregivers in exchange for respite time

The proportion of institutions with a positive opinion on partnership with caregivers was high: 74.7% (95% CI: 64.8–83.1%).

#### Type of partnership by type of institution

The comparison between non-medical and medical facilities shows differences in various types of partnerships that could be offered to caregivers.

Medical institutions would be better able to offer more organizational and governance-type activities (ethics commissions and reception of newcomers or collaborators) than non-medical institutions. Medical facilities would potentially be able to offer more care activities (care seminars, patient/family needs assessments, care plans, coordination of care and health education) as well as activities in connection with service offers (entertainment, socio-cultural activities, practical help, support and meals) than non-medical establishments. Non-medical institutions would be better able to offer training activities (simulation-based courses, practical training, initial and postgraduate training, peer training) or research activities (co-construction of research protocols, creation of scientific days) than medical institutions (see Table 2). In their comments, the participants described other partnership ideas, such as the participation of caregivers in health homes, establishment of caregivers’ cafes or permanence for caregivers. Their participation also planned to host families caring for patients with chronic diseases (see Table 2).

**Table 2:** Type of partnership by type of institution: * survey variables

Type of partnership	Type of institution		*P*-value
	Non-medical, *n* (%)	Medical, *n* (%)	
Proposals for organizational and governance activities*			
Ethics committee	3 (17.7)	**44 (56.4)**	0.004
Management fee	2 (11.8)	4 (5.1)	0.308
Commission to support a specific population	7 (41.2)	38 (48.7)	0.573
Welcoming newcomers/patients	2 (11.8)	**30 (38.5)**	0.035
Welcoming new employees	1 (5.9)	**23 (29.5)**	0.042
None	**9 (52.9)**	11 (14.1)	< 0.001
Activities related to the provision of care*			
Nursing care	4 (23.5)	23 (29.9)	0.601
Care seminars	2 (11.8)	**44 (57.1)**	0.001
Patient/family needs assessment	3 (17.7)	**59 (76.6)**	< 0.001
Implementation of patient/family care plans	4 (23.5)	**52 (67.5)**	0.001
Care coordination	1 (5.9)	**40 (52.0)**	0.001
Analysis of complex care practice	5 (29.4)	17 (22.1)	0.518
Announcement of a diagnosis	3 (17.7)	18 (23.4)	0.608
Health education/therapeutic education	6 (35.3)	**50 (64.9)**	0.024
None	**9 (52.9)**	2 (2.6)	< 0.001
Activities related to the service offer*			
Animations	3 (17.7)	**42 (53.9)**	0.007
Socio-cultural	1 (5.9)	**38 (48.7)**	0.001
Practical help	2 (11.8)	**37 (47.4)**	0.007
Accompaniment (race...)	3 (17.7)	**54 (69.3)**	< 0.001
One-time call for help	3 (17.7)	25 (32.1)	0.238
Meal provision	0 (0)	**32 (41.0)**	0.001
None	**10 (58.8)**	9 (11.5)	< 0.001
Activities related to training*			
Learning through theoretical education	14 (82.3)	46 (59.0)	0.070
Learning through practical teaching	13 (76.5)	46 (59.0)	0.178
Learning through teaching using simulation	**14 (82.4)**	30 (38.5)	0.001
Learning through practical internships	**10 (58.8)**	15 (19.2)	0.001
Initial training for carers	**13 (76.5)**	24 (30.8)	< 0.001
Postgraduate training for carers	**11 (64.7)**	16 (20.5)	< 0.001
Peer training	**10 (58.8)**	21 (26.9)	0.011
None	0 (0)	12 (15.4)	0.084
Research activities*			
Co-construction of research protocols	**8 (47.1)**	18 (23.1)	0.044
Fundraising for the project	4 (23.5)	14 (18.0)	0.595
Request to the ethics committee	4 (23.5)	13 (16.7)	0.504
Research coordination sessions	4 (23.5)	11 (14.1)	0.334
Participation of scientific days	**13 (76.5)**	15 (19.2)	< 0.001
None	3 (17.7)	**41 (52.6)**	0.009
Accompaniment and support activities*			
Support sessions	9 (52.9)	49 (62.8)	0.449
Advice to patients/families	10 (58.8)	58 (74.4)	0.198
Coordination/orientation of care pathways	6 (35.3)	34 (43.6)	0.530
Help with administrative procedures	5 (29.4)	38 (48.7)	0.147
None	**7 (41.2)**	10 (12.8)	0.006

#### Type of respite granted to caregivers in exchange for their skills

A comparison of the two types of facilities indicated differences in home respite devices: non-medical facilities are more likely to offer day respite, while medical facilities may offer night respite and combined day/night respite. Overall, non-medical institutions could more frequently consider offering training in exchange for the skills of caregivers compared to medical facilities. Medical institutions may consider administrative support, psychological counselling or home-based meals more often than non-medical institutions (see Table 3).

**Table 3:** Type of respite available to caregivers in exchange for their skills. * survey variables

Type of respite	Type of institution		*P*-value
	Non-medical, *n* (%)	Medical, *n* (%)	
Favourable opinion regarding the provision of a free respite arrangement*	14 (82.4)	56 (71.8)	0.370
Home respite plan that can be offered to family caregivers in exchange for their skills*			0.031^a^
Daytime respite	**8 (47.1)**	14 (18.0)	
Night-time respite	0 (0)	**5 (6.4)**	
Day and night respite	4 (23.5)	**42 (53.9)**	
None	5 (29.4)	17 (21.8)	
Residential respite program UATR (temporary respite care unit) can be offered to family caregivers in exchange for their skills*	2 (11.8)	8 (10.3)	0.337^a^
Daytime respite	0 (0)	1 (1.3)	
Night-time respite	9 (52.9)	56 (71.8)	
Day and night respite	6 (35.3)	13 (16.7)	
None			
Respite in the community can be offered to caregivers in exchange for their skills*			0.797^a^
Daytime respite	2 (11.8)	12 (15.4)	
Night-time respite	0 (0)	1 (1.3)	
Day and night respite	9 (52.9)	44 (56.4)	
None	6 (35.3)	21 (26.9)	
Combined respite plan that can be offered to family caregivers in exchange for their skills*			0.271^a^
Daytime respite	3 (17.7)	10 (12.8)	
Night-time respite	0 (0)	0 (0)	
Day and night respite	6 (35.3)	44 (56.4)	
None	8 (47.1)	24 (30.8)	
Possibility of offering other benefits than respite to family caregivers in exchange for their skills*	12 (70.6)	49 (62.8)	0.545
Other possible compensation*			
Coordination of the care pathway	4 (23.5)	17 (24.6)	0.599^a^
Training offer	**14 (82.4)**	34 (49.3)	0.014
Administrative support	2 (11.8)	**28 (40.6)**	0.026
Psychological follow-up	3 (17.7)	**30 (43.5)**	0.050
Meals at home	1 (5.9)	**25 (36.2)**	0.015
Remote monitoring subscription	1 (5.9)	18 (26.1)	0.103^a^
None	3 (17.7)	13 (18.8)	0.999^a^
Existence of a win/win partnership mechanism with caregivers in the institution*	0 (0)	5 (6.4)	0.581^a^

^a^Fischer’s exact

In the comments, the participants identified win-win partnership arrangements, but the situations described did not provide specific compensation for the needs of the caregiver (see Additional file 2).

### Synthesis of the results (see Figs. 1, 2, 3, 4, 5 and 6)

Executives of health-related institutions in the canton of Geneva are in favour of win-win partnerships with caregivers. Partnership offers were linked to activities of institutions based on organization, governance, care, service, accompaniment, support, training and research in return for a free respite device.

**Fig. 1:**
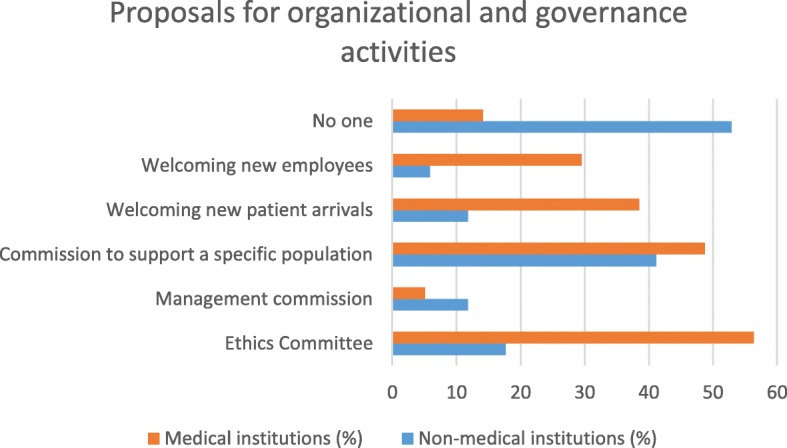
Type of partnership in the area of organization/governance by type of institution

**Fig. 2:**
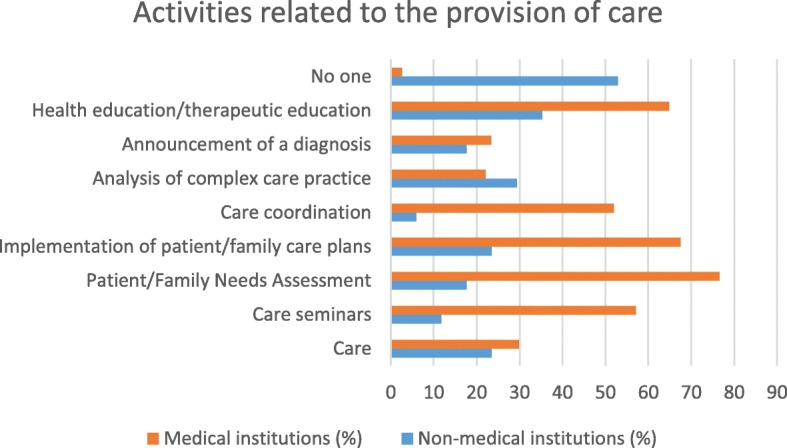
Type of partnership in the field of care according to type of institution

**Fig. 3:**
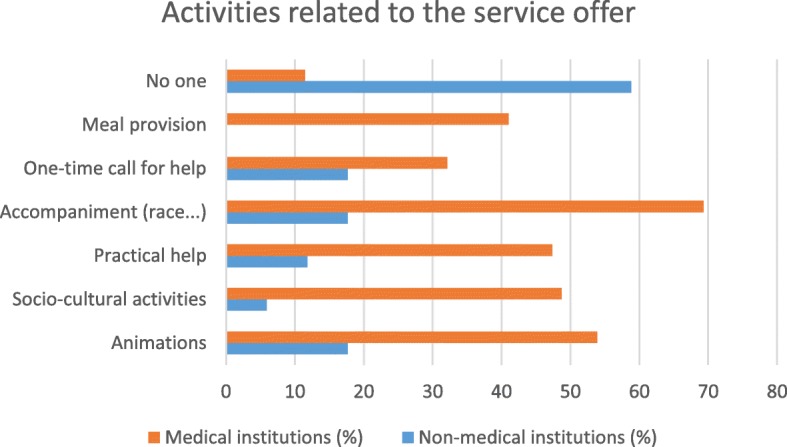
Type of partnership in the field of service offering by type of institution

**Fig. 4:**
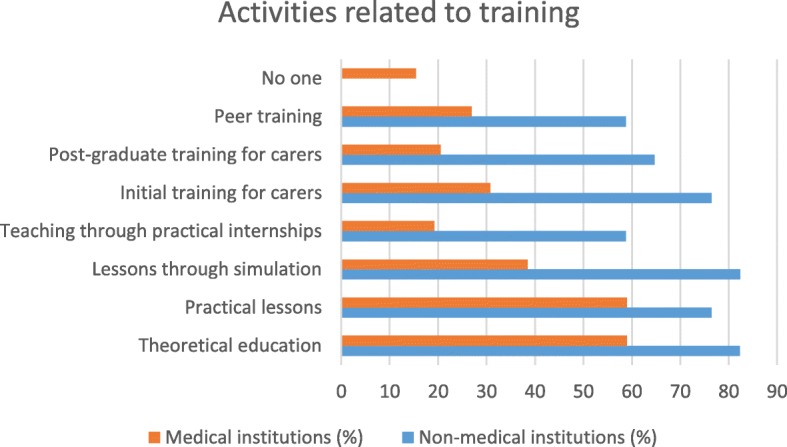
Type of partnership in the field of training according to type of institution

**Fig. 5:**
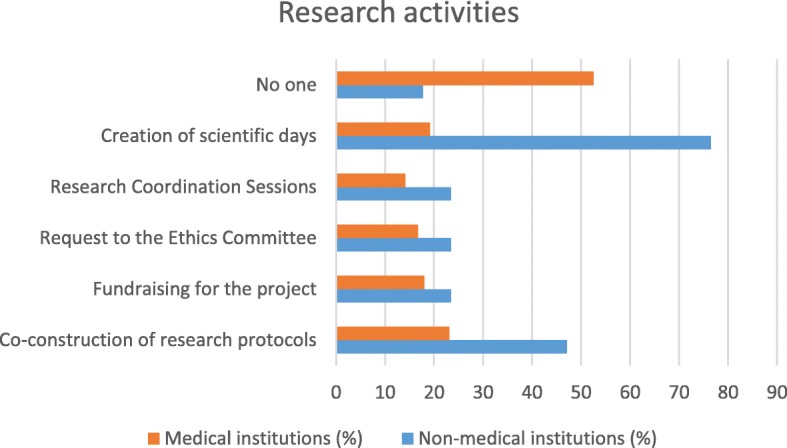
Type of partnership in the field of research according to type of institution

**Fig. 6:**
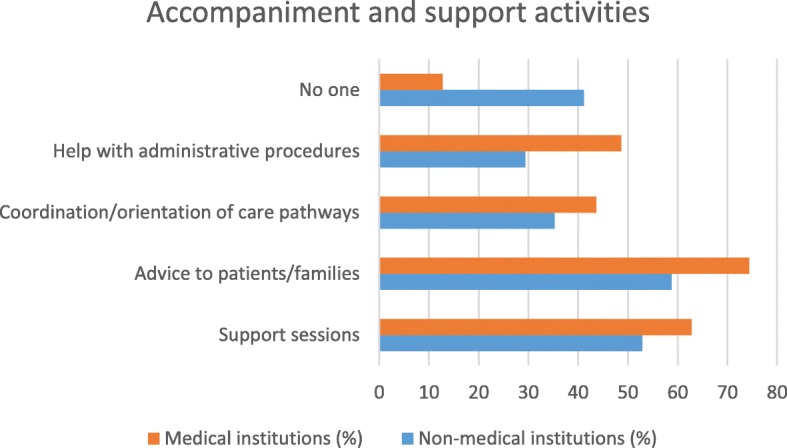
Type of partnership in the field of accompaniment/support according to type of institution

There are differences in the partnership offers between medical institutions and non-medical institutions. Organizational and governance-type activities and care and service offerings are more likely to be offered by medical facilities. Non-medical institutions were better able than other institutions to offer partnerships around training and research. The most significant results (*p* <  0.001) were identified in partnerships between institutions related to healthcare and family caregivers when setting up patient/family care plans and accompanying other caregivers. On the other hand, with regard to health-related but non-medicalised institutions, partnerships with very significant results (*p* <  0.001) were identified for activities related to initial and postgraduate training for caregivers and the creation of scientific study days.

The compensation offered in exchange for caregivers skills differs between the two types of establishments. Day respite could be offered by non-medical facilities, while night respite and combined day/night respite could be offered by medical facilities. In addition, medical and non-medical institutions could eventually offer other types of compensation.

## Discussion

The purpose of this study was to identify activities related to the functioning of health-related institutions that could be entrusted to caregivers in exchange for a free respite device. In a changing national context regarding home care, as well as the growing involvement of caregivers, the results of this study are promising. They outline the possibility of win/win partnerships between health-related institutions and caregivers. The caregivers involved in this study were those caring for people with dementia. However, the partnerships can be generalised to other types of caregivers. Indeed, activities that can potentially be identified as part of a possible exchange with caregivers’ skills are not specific to dementia. For example, a caregiver of a diabetic child can participate just as much as a caregiver of a person with dementia when designing a research protocol or training a peer.

The results show that both medical and non-medical institutions are positive about potential partnerships in areas such as organization, governance, care-related activities, service offerings, training, research, accompaniment and support activities. This goes in direction with the Montreal Model, which proposes patient/family involvement in the various fields mentioned above [11]. Programs based on this model have shown positive results in improving quality of practices throughout the care process (reception, diagnosis announcement, care planning, etc.) as well as in collaborative practices and at the level of the institutional culture concerning partnership with the patient. They also highlighted an added value for the patient/family in improving a sense of social utility [20]. Regarding our study, the results highlight that the proportion of institutions with a favourable opinion towards partnership with caregivers is high: 74.7% (95% CI: 64.8–83.1%).

However, according to studies conducted by Carman et al. in 2013 [21] as well as the Center for Applied Pedagogy in Health Sciences at the University of Montreal in 2014 [22], several factors can influence the sustainability of a partnership program between caregivers, patients and their families: the characteristics of patients/relatives (values, attitudes, experiences, etc.); institutional culture and social norms that could influence the commitment of caregivers, the remuneration of various actors of the project and the training of patients/families as well as health professionals.

Joint training is envisaged by some universities to promote partnership between caregivers, patients and relatives [20, 21]. The partnership is designed to share scientific knowledge of professionals and experiential knowledge of patients/families acquired during their life course with the disease [22].

The aim is to integrate patients/families within the health system as learners but also as bearers of experiential knowledge which allows health-related institutions to be learners as well [17]. The ultimate goal of this approach is to improve quality of life for the patient/family, as well as quality of care in coordination, safety, accessibility and efficiency [17]. Indeed, patients/families mobilize their expertise in relation to their experiences and not a scientific expertise held by various members within a health institution. However, when the latter are too far removed from the problems and needs of citizens, their actions tend to focus on their own issues [14]. For a decade, several initiatives have been proposed at the international level in the United States [15], Great Britain [16] and France [12, 17]. Patient/family involvement initiatives concerning decisions and functioning of health-related institutions remain, according to Charpak, anecdotal to needs [14]. As highlighted by some authors, lack of recognition can be a barrier to partnership [20, 21]. Indeed, according to Honneth and Rusch, the recognition of society for services rendered is paramount for the psychological stability of citizens [19]. This is the case for family caregivers who, in 2014, thanks to their activities, saved Switzerland’s healthcare system 3.4 billion CHF in costs. As a result, the Hestia program is considering recognition through a no-cost response for the needs of caregivers. In this context, the results of this study were able to highlight examples of compensation envisaged to meet the recognition needs of caregivers. Indeed, this compensation has answered various needs which are abundantly identified in the literature [9]. Family caregivers essentially need respite, training, information, recognition, financial aid as well as to fight against social isolation [8, 9]. The proposed compensation forms mainly involve the provision of training, administrative support, organizational support, psychological follow-up and a free respite device. Between the needs identified in the literature and the compensation offers envisaged in this study, we can see responses to needs. In fact, taking the example of respite, the results show high interest in proposing a free respite device in exchange for the skills of caregivers (73.7%; 95% CI: 63.6–82.2%). The different types of respite offered respond to an existing need for respite both day and night.

However, it is important to stay vigilant towards the vulnerability of caregivers. They are among the main actors of the health system. They help to promote home support of their loved ones. Their involvement is strong, but in the long run, can be exhausting for some. It favours the onset of feeling of burden with psychological and physical exhaustion. This state is accentuated by social isolation, lack of recognition and lack of respite. To combat this, according to Meleis in 2010, it is essential to accompany the caregiver in his or her role change, particularly in the acquisition of knowledge and skills not yet mastered; in his or her ability to adapt; in the sense given to his actions; in feeling connected; in constant interaction; in his or her experience with illness; in developing his or her confidence and in becoming aware of his or her new role [23]. As part of a win/win partnership project, it is hypothesized that recognition as a direct response to caregivers’ own needs can only improve their transition. Given the risk of burnout, it is therefore important to think about projects which take into account the wishes and availability of caregivers. This activity must in no case be obligatory for caregivers; it must be voluntary and respond in a systemic and unitary way to their needs and expectations.

## Limitations and interests of the study

The strength of this study is in the local evaluation of potential partnerships that can provide a response to local policies that are, among other things, focused so far on the support of caregivers [24]. It will allow for the possibility of win/win partnerships. It focused on the population facing dementia but the results are transferable to other populations. This study had some limitations. The survey was conducted with health-related institutions; however, it could also have been carried out with institutions related to the social environment or industry. Indeed, the issues of caregivers also fall within these two areas. The partnership proposals could have been even richer and more diversified. In addition, the survey used in this study aimed to sound out the proportion of positive opinions for a partnership between health-related institutions and caregivers in the canton of Geneva. In addition, the goal was also to identify local health-related activities that could be potential partners. Such a survey did not exist. It was therefore created specifically for the study. The tests performed to verify the validity of this survey correspond to the content validity tests described by Fortin M. F in 2006. The psychometric tests to verify the fidelity of this instrument have not been performed and represent a limit to this study.

## Conclusion

This study shows that the senior staff of health-related institutions in the canton of Geneva are interested in establishing win/win partnerships with caregivers of people facing dementia. These positive results encourage new partnerships. In the future, innovative projects can emerge to meet the needs of each party. The caregiver’s position can evolve to a position that will give him or her the right to use his or her skills in institutions related to health. Caregivers will be considered even more as actors of public utility. The care will not only be thought of as being centred on the patient’s/family’s health concerns and problems but also in partnership with the patient/family. This is a paradigm shift that has already evolved across the Atlantic but must make its mark at the European level.

## Additional files

Additional file 1: Questionnaire, potential partnerships between health-related institutions and caregivers. (DOCX 94 kb)

Additional file 2: Participants’ answers to the open-ended questions of the questionnaire. (DOCX 46 kb)

## Data Availability

All data used and/or analysed in the current study are available from the corresponding author upon reasonable request.
