# Supplementary material for: Win/win partnerships between Geneva health-related institutions and caregivers of people with dementia: a descriptive cross-sectional study
Source: BMC Public Health. 2019 Jun 7;19:714. doi: 10.1186/s12889-019-7014-8 (PMC6556013; doi:10.1186/s12889-019-7014-8)
Supplement: Supplementary file 1 — Questionnaire, potential partnerships between health-related institutions and caregivers. (DOCX 94 kb) [file 12889_2019_7014_MOESM1_ESM.docx]

**THE DESIGN OF AN ANONYMOUS IDENTIFICATION NUMBER**

**In order to make your participation anonymous, the following 5 questions will generate a number that will allow statistical monitoring of the data without being able to identify you.**

1. **Which is the last letter of your first name?**

A B C D E F G H I J K L M N O P Q R S T

U V W X Y Z

1. **Which is the first letter of your neighbour's first name?**

A B C D E F G H I J K L M N O P Q R S T

U V W X Y Z

1. **What is your zodiac sign?**

Aries Taurus Gemini Cancer Leo Virgo Libra Scorpio

Sagittarius Capricorn Aquarius Pisces

1. **What day is it today?**

1 2 3 4 5 6 7 8 9 10 11 12 13 14 15 16 17 18

19 20 21 22 23 24 25 26 27 28 29 30 31

1. **What is your lucky number?**

1 2 3 4 5 6 7 8 9 10 11 12 13 14 15 16 17 18

19 20 21 22 23 24 25 26 27 28 29 30 31

**THE SOCIO-DEMOGRAPHIC DATA OF THE INSTITUTION WHERE YOU WORK**

1. **Name of your organization:**

_1_ Medical and social establishment

_2_ Home care facility

_3_ Training Institute

_4_ General Directorate of Health

_5_ Patient and family association

1. **What is the status of your establishment?**

_1_ Public _2_ Private

1. **Among the following choices, check the main mission that applies to your institution:**

_1_ Education / training

_2_ Care and services

_3_ Health system management and coordination

_4_ Support, advice and guidance

**DESCRIPTION OF THE ACTIVITIES OF THE INSTITUTION WHERE YOU WORK**

1. **Which of the following activities are currently present in your institution?**
2. *Organizational activities and governance:*

_1_ Ethics Committee

_2_ Management fee

_3_ Commission to support a specific population

_4_ Activities to welcome new patient arrivals

_5_ Activities to welcome new employees

_6_ Other (complete below) ……………………………………………………………………………………………………………………………………………………………………………………………………………………………………………………………………………………………………………………………………………………………………………………………………………………………………………………………………………………………………………………………………………….……

1. *Activities related to the provision of care*

_1_ Respite care

_2_ Nursing care

_3_ Care conferences

_4_ Assessment of the needs of patients and/or families

_5_ Implementation of the care plan for patients and/or families

_6_ Care coordination

_7_ Analysis of complex care practice

_9_ Announcement of a diagnosis

_10_ Health education/patient-oriented therapeutic education and/or family education

_11_ Other (complete below) ……………………………………………………………………………………………………………………………………………………………………………………………………………………………………………………………………………………………………………………………………………………………………………………………………………………………………………………………………………………………………………………………………………………

1. *Activities related to the provision of services*

_1_ Animation activities

_2_ Socio-cultural activities

_3_ Practical help

_4_ Assistance (groceries, appointments etc.....)

_5_ A single telephone number for assistance, guidance and support

_6_ Meal provision

_7_ Other (complete below) ………………………………………………………………………………………………………………………………………………………………………………………………………………………………………………………………………………………………………………………………………………………………………………………………………………………………………………………………………………………………………………………………………………….…

1. *Activities related to training*

_1_ Learning through theoretical instruction (group and individual)

_2_ Learning through practical lessons (technical workshops)

_3_ Learning through teaching through simulation (use of standardized patients and high-fidelity simulation)

_4_ Learning through practical internships

_5_ Learning through patients and/or expert families

_6_ Initial training of carers

_7_ Post-graduate training for carers

_8_ Other (complete below) ……………………………………………………………………………………………………………………………………………………………………………………………………………………………………………………………………………………………………………………………………………………………………………………………………………………………………………………………………………………………………………………………………………………

1. *Research-related activities*

_1_ Design of research protocols

_2_ Search for third-party funds

_3_ Official request to the Ethics Commission

_4_ Research Coordination Sessions

_5_ Creation of scientific days

_7_ Other (complete below) ………………………………………………………………………………………………………………………………………………………………………………………………………………………………………………………………………………………………………………………………………………………………………………………………………………………………………………………………………………………………………………………………….…………………

1. *Activities related to accompaniment and support*

_1_ Support session (focus groups, individual support)

_2_ Advice to patients and/or families

_2_ Coordination/orientation of patient care pathways

_3_ Help with administrative procedures

_4_ family relief/respite

_5_ Other (complete below) ………………………………………………………………………………………………………………………………………………………………………………………………………………………………………………………………………………………………………………………………………………………………………………………………………………………………………………………………………………………………………….…………………………………………

1. **Do you currently have any devices, activities or sessions for caregivers?**

_0_ No _1_ Yes

1. If yes, how many do you have?

_0_ 0 _1_1 _2_2 _3_3 _4_4 _5_5 _6_6 _7_7 _8_8 _9_9 _10_10 _11_˃10

**PERCEPTIONS OF A POSSIBLE PARTNERSHIP WITH FAMILY CAREGIVERS**

1. **Would you be open to possibly being able to assign activities from your institute to caregivers?**

_0_ No _1_ Yes

1. Using the choices below, identify the statement that best reflects your opinion about this possibility.

_0_ Do not agree at all_1_ Rather disagree_2_ Pretty much agree_3_ Fully agree

1. **Among the activities that already exist, identify those that could potentially be entrusted to caregivers in your institution:**
2. *Organizational activities and governance:*

_1_ Participation in an ethics committee

_2_ Participation in a management commission

_3_ Participation in a commission to support a specific population

_4_ Participation in welcoming newcomers/patients

_5_ Participation in activities to welcome new employees

_6_ Other (complete below) ………………………………………………………………………………………………………………………………………………………………………………………………………………………………………………………………………………………………………………………………………………………………………………………………………………………………………………………………………………………………………………………………………………………………………………………………

1. *Activities related to the provision of care*

_2_ Participation in nursing care

_3_ Participation in care seminars

_4_ Participation in the needs assessment of patients and/or families

_5_ Participation in the implementation of patient and/or family care plans

_6_ Participation in the coordination of care

_7_ Participation in the analysis of complex care practice

_8_ Participation in the announcement of a diagnosis

_9_ Participation as an expert caregiver in health/therapeutic education of a patient and/or family

_10_ Other (complete below) ………………………………………………………………………………………………………………………………………………………………………………………………………………………………………………………………………………………………………………………………………………………………………………………………………………………………………………………………………………………………………………………………………………………………………………………………

1. *Activities related to the provision of services*

_1_ Participation in animation activities

_2_ Participation in socio-cultural activities

_3_ Participation in practical assistance

_4_ Participation in support services (groceries, appointments, etc.)

_5_ Participation as a resource in the single help, guidance and support number

_6_ Participation in the provision of meals

_7_ Other (complete below) ………………………………………………………………………………………………………………………………………………………………………………………………………………………………………………………………………………………………………………………………………………………………………………………………………………………………………………………………………………………………………………………………………………………………………………………………

1. *Activities related to education and training*

_1_ Participation in theoretical training as expert caregivers

_2_ Participation in practical lessons as expert caregivers

_3_ Participation as simulated patients for simulation activities

_4_ Participation in learning through practical internships at home

_5_ Participation in initial training for carers

_6_ Participation in post-graduate training in health care

_7_ Participation in peer training

_8_ Other (complete below) ………………………………………………………………………………………………………………………………………………………………………………………………………………………………………………………………………………………………………………………………………………………………………………………………………………………………………………………………………………………………………………………………………………………………………………………………

1. *Research-related activities*

_1_ Participation in the co-construction of research protocols

_2_ Participation in the search for third-party funds

_3_ Participation in the official request to the ethics commission

_4_ Participation in research coordination sessions

_5_ Participation in the creation of scientific days

_6_ Other (complete below) …………………………………………………………………………………………………………………………………………………………………………………………………………………………………………………………………………………………………………………………………………………………………………………………………………………………………………………………………………………………………………………………………………………………………………………………..…

1. *Activities related to accompaniment and support*

_1_ Participation in the facilitation of support sessions (focus groups, individual support)

_2_ Participation in support sessions (focus groups, individual support)

_3_ Participation in the counselling system provided to patients and/or families

_4_ Participation in the coordination/orientation of patient care pathways

_5_ Participation in assistance with administrative procedures

_6_ Other (complete below) ……………………………………………………………………………………………………………………………………………………………………………………………………………………………………………………………………………………………………………………………………………………………………………………………………………………………………………………………………………………………………………………………………………………………………………………………..

1. **How often do you think it is feasible and possible to offer participation in activities to caregivers?**

_0_ 0 _1_1 _2_2 _3_3 _4_4 _5_5 _6_6 _7_7 _8_8 _9_9 _10_10 _11_˃10 activities /week

_0_ 0 _1_1 _2_2 _3_3 _4_4 _5_5 _6_6 _7_7 _8_8 _9_9 _10_10 _11_ ˃10 activities /month

_0_ 0 _1_1 _2_2 _3_3 _4_4 _5_5 _6_6 _7_7 _8_8 _9_9 _10_10 _11_ ˃10 activities /year

1. **Are you open to the possibility of providing a caregiver with a free respite arrangement in exchange for their skills?**

_0_ No _1_ Yes

1. **How many respite care/replacement devices are there in your institution (all devices that ensure that patients' care is taken over (e.g., monitoring, parental substitution, etc.) are included in respite systems?**

_0_ None

_1_ 1 respite device

_2_ 2 respite device

_3_ 3 respite device

_4_ More than 4 respite devices

1. **Among the choices listed below, check the respite device(s) that could be considered and offered to caregivers in exchange for their skills.**
2. Home respite care

_1_  Daytime respite _2_ Night respite

1. Residential respite facility UATR (Temporary Respite Reception Unit, others)

_1_  Daytime respite _2_ Night respite

1. Respite in the living environment (EMS, others)

_1_  Daytime respite _2_ Night respite

1. Combined respite (patient and caregiver care)

_1_  Daytime respite _2_ Night respite

_3_ Other (complete below) ………………………………………………………………………………………………………………………………………………………………………………………………………………………………………………………………………………………………………………………………………………………………………………………………………………………………………………………………………………………………………………………………………………………………………………………………

1. **Is it possible to imagine that your institution would be willing to evaluate the possibility of being able to offer other benefits than respite to family caregivers in exchange for their skills?**

_0_ No _1_ Yes

1. If yes, tick the compensation(s) for which you would be open to offer to caregivers:

_1_ Offering coordination of the patient's care path

_2_ Provision of training

_3_  Providing administrative support

_4_  Offer of psychological follow-up

_5_ Meals on Wheels Offer

_6_ Offering practical help at home

_7_ Offer of a remote monitoring subscription

_8_ Other (complete below) ………………………………………………………………………………………………………………………………………………………………………………………………………………………………………………………………………………………………………………………………………………………………………………………………………………………………………………………………………………………………………………………………………………………………………………………………

1. **Do you have any other ideas about possible partnerships that could exist between caregivers and health-related institutions?**

………………………………………………………………………………………………………………………………………………………………………………………………………………………………………………………………………………………………………………………………………………………………………………………………………………………………………………………………………………………………………………………………………………………………………………………………

Thank you for your participation!

If you have any questions about the survey, please feel free to write to us at the following address: marie.leocadie@hesge.ch
