# Supplementary material for: Win/win partnerships between Geneva health-related institutions and caregivers of people with dementia: a descriptive cross-sectional study
Source: BMC Public Health. 2019 Jun 7;19:714. doi: 10.1186/s12889-019-7014-8 (PMC6556013; doi:10.1186/s12889-019-7014-8)
Supplement: Supplementary file 2 — Participants’ answers to the open-ended questions of the questionnaire. (DOCX 46 kb) [file 12889_2019_7014_MOESM2_ESM.docx]

Additional file 2: Participants' answers to the open-ended questions of the questionnaire.

| **Questions** | **Answers** | **Summary** |
| --- | --- | --- |
| Identify other activities in your institution that are not mentioned in the proposals made previously (complete opposite, if applicable) | - « patient safety (remote alarm) Occupational therapy 24-hour response» - « updating knowledge and skills (workshops, protocols, care techniques, guidelines and recommendations), expert consultation activities, dietician and occupational therapy consultations, interventions in continuing education (Heds, HUG) » - « updating of knowledge (workshops, protocol, techniques, care guidelines and recommendations), expert consultation activities Dietary consultation and occupational therapy Interventions in continuing education HEDS/HUG» - « health of catamnesis personnel» - « nutritional counselling emergency social support» - « lessons learned: clinical evaluation simulations blended learning...» - « emergency intervention for disadvantaged populations, the most vulnerable, social emergencies» - « participation in research as an expert. Participation in network days (network conference, inter-Ems days...) and in various continuing education spaces as adult trainer, HEDS / FEGEMS spaces..., services...» - « provision of specific teaching services» - « participation in research protocols» - « accompaniment in palliative care. (clinical nurse and internal and external training). Internal training in good treatment (MRPS has received the Sonata label). Member of Fegems (participation in the Fegems Ethics Committee / training programmes etc.). Social worker in the institution. Personalized assistance for the transition from our residence to the EMS part (depending on the evolution of the dependency). Falls Monitoring Group (prevention plan). Internal home care service in the "Residence" section. Participation in the Petit-Saconnex Neighbourhood Platform. Intergenerational. Activities (with Pt Saconnex nursery and school of Crêts). - « for more details: travel accompaniment - meal preparation (cooking) - intervention with the paediatric-adult and geriatric population, also with the population with disabilities (physical and mental). Collaboration with the Protection of Minors to set up a framework allowing home care and avoiding home for minors». - « planning appointments for external workers Accompaniment of residents for systematic appointments if family carers cannot do so Creation of a training platform using filmed sequences on a voluntary basis and agreement». | Participants in the questionnaire listed a multitude of secondary activities in their institution. |
| Forward question] (Would you be open to assigning activities from your institution to caregivers? Identify, using the choices on the left, the statement that best reflects your opinion about this possibility.)  If you do not agree at all, please inform us why? | - « in partnership» - « caregivers are already under great pressure, health professionals are not there to overload them but to help and relieve them, each in their own place! You can't replace professional skills without high-level training». - « we are here to relieve them precisely... For their participation, some want to learn and be autonomous (for example in the management of a SAD)» - « mixed opinion: -Involving the caregiver more can be stimulating for him/her, opening up to other activities and better understanding the patient's situation, and reducing health costs, after training and/or education - at the same time, entrusting even more activities to the caregiver, can be a source of exhaustion, and responsibilities». - « the caregiver is often exhausted and hospitalization can allow him or her to take care of himself or herself, to take care of the person differently. It is often important that they hear that they have the right to rest, that it is important that they take it from him too. Beware of the guilt he might feel if he was offered activities to do with his loved one, and that he would not dare to say no. Each situation is to be assessed I think » - « the hospital is a place where the next caregiver can rely on professional support. A moment of respite for him» - « accompaniment to appointments, meal assistance». - « the purpose of our services is to relieve the caregiver by taking care of the loved one being cared for. I can't imagine delegating part of our services to him. As the caregiver is our main representative, he or she already participates automatically in the coordination and information aspect ». | There are four main areas of opposition to the fact that caregivers are responsible for the institution's activities:  - for the most part, this would be a source of over solicitation for the caregiver, leading to more responsibilities. These causing more exhaustion.  - delegating tasks to the caregiver would not provide him or her with the respite he or she needs by entrusting the sick person to institutions.  - caregivers are not considered to have the same skills and training as professionals.  - for a person this can be accepted but only in partnership. |
| Identify other possible caregiver involvement within your institution (complete opposite, if applicable) | - « participation of family carers in the animation within the IEPA building with supervision for elderly people». - « testimony » - « catamnesis » - « testimonies, that's already the case. To be reinforced» - « participation in educational groups to provide support upon return home» - « participation in our residents' outings». - « caregivers can participate in welcoming relatives when they enter an institution». | The participation of caregivers in institutions could be done by:  - collaboration in the institution's activities  - support for outings  - training through the testimonies of the caregiver  - assistance in the preparation of nursing files (catamnesis, reception at the entrance). |
| Comments on proposals for the participation of family caregivers in institutions (complete opposite, if applicable) | - « the caregivers we meet in our work are very often exhausted; asking them to participate in institutional activities seems to me difficult to imagine and could increase their "burden".». - « you must first identify the caregiver's benevolence or malevolence (e.g., attention to a caregiver with unhealthy financial interests)» - « caregivers could integrate the majority of the institution's activities if they are provided with knowledge, skills and spaces for support, debriefing, etc.» - « a caregiver may have difficulty finding time to get involved in a role in addition to supporting their loved one. It seems to me that they need more respite at first. Perhaps see the possibility of valuing their experience and skills when they came out of this important support? » - « contribution to be strengthened, taking into account the expertise developed by family caregivers.» - « as a facilitator of a training course for Caregivers of Persons with Dementia in 2014 and 2015, I found that family members were not very available and were very much in demand in their role. » - « the presence of family caregivers in the process of comprehensive patient care is essential today.» - « this is a rapidly expanding field, but I do not know how my entire institution works.» - « i think it would be best to put the families of new residents in contact with family caregivers. » - « for me, family caregivers can participate in everything within our institution, as long as it is their desire and as long as we can also support them to have a break and mobilize external resources to enable them to keep a social life outside of the family member living with us. » | There are divergent opinions in the comments regarding the participation of family caregivers in institutions.  The caregiver's lack of time can hinder his or her investment. Assigning institutional activities and a role could lead to an over solicitation of the caregiver, leading to exhaustion and an increase in the burden he or she has. A person wonders about the caregiver's willingness to be caring and malicious.  However, the participation of the caregiver remains essential in supporting the care of the patient as a whole. The expertise and skills learned can be valued by institutions.  In return for the caregiver's participation, institutions could provide them with knowledge, skills, debriefing support and respite time to enable them to maintain a social bond. |
| Comments on your opinion as to the possibility of giving the family member a free respite in exchange for his or her skills: (complete opposite, if applicable) | - « a respite is used so that the caregiver can breathe. I don't think he can invest more than he already does in his own home, but the idea may have to be explored. A remuneration for the caregiver and a reduction in the UATR rate would be more appropriate.» - « enhancement of the role and financial value of the activities carried out by the caregiver. » - « if the carer is involved on a daily basis with a relative at home, he/she can limit hospitalizations and care services... the carer could therefore benefit from a respite period without additional costs. » - « it seems to me that this could be moved in some cases where the caregiver may feel compelled to receive assistance in return. Why not value the loved one's expense without compensation?» - « i agree that the beneficiary may give his or her time to compensate for the free nature of the scheme. It is just necessary to frame the time by avoiding imposing too much presence on the beneficiary and exhausting him more. Basically, the system is designed to give them a break.» - « respite for the UATR family type offered as compensation?» - « for the time being we have not thought about or considered proposal 1.29 but this point should be studied. » - « as mentioned in the title of the study, this would be a "win-win" situation: participating in the care of the client and having the opportunity to "deposit" the overflow of emotions, seeking solutions, getting to know the network and the help, listening to the experiences of other caregivers, etc...» - « recognition and financial support. » - « a system that gives/give training for time in exchange for the skills of caregivers» - « this person assumes tasks that are necessary and should be delegated to professionals to support the person who needs them. As a result of this unpaid activity, the company'saves money'. It seems to me that it is a normal return to consider free respite service. » - « this gives me the impression that not only does he already do a lot for his loved one, but he must also give of himself to society to obtain a benefit, while the fact of paying allows him a real break. For some people, this can be a recognition. Be careful of the risk of total exhaustion of family caregivers.» - « for me, it is an exchange of good processes, in the sense that the activities represent a different motivation (going beyond the framework of a caregiver, putting one's experience at the service of others, exchanges with others) The free respite system would make it possible to take a "break" and to enhance it» - « the question remains of funding.» - « what's respite?» - « no respite arrangements within the institution and other external arrangements that are often overloaded» - « the hospital is a place of acute care and should not replace the duty of society to create respite places, which do not exist in Geneva (no one wants these heavy patients). I am thinking in particular of the chronically ill, patients with various dementias, neurological diseases (SLA...)» - « i am not a decision-maker in this area.» - « we do not offer short stay accommodation such as UATR.» - « being an EMS, the respite is there...» - « as a private company with no refund, we do not consider this type of service.» | The questions that arise are whether the caregiver has enough time to do more than he or she already does? Wouldn't he like to pay for a real moment of respite? Wouldn't he feel compelled to give his help to access a device at no cost? Isn't providing help going to lead to total exhaustion?  Most current respite arrangements are not available. In addition, hospitals should not be substitutes for the lack of respite facilities.  Nevertheless, most participants highlighted the money savings that family caregivers can bring by taking care of their loved one (fewer hospitalizations, fewer services, etc.). They therefore all agree that by exchanging their skills, a free scheme could be granted to them. However, the time allocated to the institution should not exceed. One participant also suggested paying caregivers and lowering the fees for UATRs. |
| Other respite options that could be offered to the caregiver? (complete opposite, if applicable) | - « service around a table dedicated to the caregiver 1x/week with a group discussion afterwards» - « lunch at lunchtime in the IEPAs» - « respite in care and practical assistance» - « customer/caregiver holiday» - « patient-caregiver holidays» - « baling» - « holidays with their sick relatives and carers as does the Alzheimer association. Home'baluchoning' for people who need to stay in their environment.» - « UATRs only receive stable patients who are difficult in these diseases....» - « meals » - « i do not fully understand these questions about respite. For me, the principle of residing in a HSF means that the caregiver can give what he or she wants because everything can be done by the HSF staff. » | Other respite options available to caregivers include the following:  - meals  - baling  - holidays for caregivers/ sick relatives  - discussion group followed by a performance around a table |
| Other possible compensations for caregivers: (complete opposite, if applicable) | - « participation in the auxiliary means put in place» - « offer them time for themselves by keeping their loved one while they are there. » - « financial offer» - « home attendance Subscriptions» - « home presence» - « shared physical activities (e. g. Nordic walking, swimming, yoga, dancing etc. with an occupational therapist) free computer courses and computer repair/update with computer specialists» - « remuneration that helps him to choose what is appropriate for him» - « partnership with HETS for assistance with the financial aspects of a social worker» - « offer of a respite time envelope at home» - « adapted yoga sessions» - « financial assistance in the event that a work stoppage is considered. » - « offer to cover the cost of additional care (shopping, cleaning)» - « to be discussed with our management and board of directors. » - « it's a track to dig!» | The possible compensation proposals are as follows:  - home help for a respite period  - remuneration / financial assistance  - assistance for financial aspects with partnership at HETS (social workers)  - health advice  - yoga sessions  - coverage of ancillary care (shopping, households)  - participation in the auxiliary means put in place |
| Do you have any other ideas about possible partnerships that could exist between caregivers and health-related institutions? | - « a true partnership and strengthening the empowerment of family members. » - « in nursing homes, at the caregivers' café,. propose a common theme for caregivers and exchange» - « have family caregivers on standby if necessary.» - « foster family for chronic patients» - « in all health-related training, a module or course should be offered during these initial training sessions.» | Possible proposals for partnerships between caregivers and institutions are as follows:  - nursing homes  - caregiver Café  - permanence for caregivers  - host family for patients with chronic diseases  - continuous training courses |
| If so, please explain the system in place in your institution: | - « concerning psychological illness, a partnership has existed for some time with meetings and mutual analysis of interventions» - « this questionnaire is very confusing and nothing is clear to the uninitiated of which I am a part.» - « vision 20/20 ? » - « THERAPEUTICS GROUP» | The "win-win" partnerships that already exist in institutions include the exchanges that families and health professionals can have when a patient with a mental illness is involved. Meetings and sharing of analysis of interventions are established.  Therapeutic groups are also considered as a partnership. |
